# Maturation-dependent expression of AIM2 in human B-cells

**DOI:** 10.1371/journal.pone.0183268

**Published:** 2017-08-15

**Authors:** Alexandra Svensson, Marianela Patzi Churqui, Kerstin Schlüter, Liza Lind, Kristina Eriksson

**Affiliations:** 1 Department of Rheumatology and Inflammation Research, Institute of Medicine, the Sahlgrenska Academy, University of Gothenburg, Gothenburg, Sweden; 2 Virology, Immunity and Infection Unit, SELADIS institute, Biochemistry and Pharmacy Faculty, Universidad Mayor de San Andres, La Paz, Bolivia; Virginia Polytechnic Institute and State University, UNITED STATES

## Abstract

Intracellular DNA- and RNA-sensing receptors, such as the IFN-inducible protein Absent in Melanoma 2 (AIM2), serve as host sensors against a wide range of infections. Immune sensing and inflammasome activation by AIM2 has been implicated in innate antiviral recognition in many experimental systems using cell-lines and animal models. However, little is known about the expression and function of AIM2 in freshly isolated human cells. In this study we investigated the expression of AIM2 in different cell types derived from human cord and adult peripheral blood, in steady state and following *in vitro*-activation. Adult but not cord blood B-cells expressed high levels of AIM2 mRNA at steady state. In adults, AIM2 was primarily expressed in mature memory CD27^+^ B-cells. Both adult and cord blood derived B-cells could induce their transcription of AIM2 mRNA in response to type II IFN but not type I IFN or the AIM2 ligand poly dA:dT. Upon B-cell receptor stimulation, B-cells from adult blood expressed reduced levels of AIM2 mRNA. In addition, we show that adult B-cells were able to release IL-1β upon stimulation with synthetic DNA. We conclude that functional AIM2 is preferentially expressed in adult human CD27^+^ B-cells, but is absent in cord blood mononuclear cells.

## Introduction

The innate human immune system is equipped with a variety of pattern recognition receptors (PRRs) that are able to sense the presence of nucleic acids. Among these are the inflammasome receptor proteins that upon binding to its ligand form the inflammasome. The most commonly discussed PPRs that are able to form inflammasomes includes the nucleotide-binding domain, leucine-rich repeat containing proteins (NLRs) and the absent in melanoma 2 (AIM2) like receptors (ALRs) (i.e. the PYHIN-family). The ALRs include the DNA sensors IFI16 and AIM2 that both recognize double stranded DNA (dsDNA) inside the cell. IFI16 can sence the presence of dsDNA in both the cytoplasm and the nucleus, whereas AIM2 sense dsDNA that is located in the cytoplasm [1–4].

Upon receptor-binding, AIM2 merge with pro-caspase-1 by its DNA-binding HIN200 domain that bind directly to the dsDNA, and the pyrin domain that allows binding to the adaptor protein ASC. In turn, the carboxy-terminal CARD of ASC binds the CARD of pro-caspase-1, which leads to the activation of caspase-1, and the subsequent formation of the AIM2 inflammasome [3,5]. The activation of caspase-1 allows for the cleavage of the cytokine precursors pro-IL-1β and pro-IL-18 into their active forms, i.e. IL-1β and IL-18. In addition to the release of pro-inflammatory IL-1β and IL-18, AIM2 inflammasome activation also leads to a lytic form of programmed cell death, which is referred to a pyroptosis [2]. The AIM2 inflammasome has been ascribed an important role in infections with a variety of pathogens, as well as in several forms of cancers and different inflammatory diseases [6]. Still, little is known about the induction and function of AIM2 in human leucocytes.

In newborns, the innate immune recognition is impaired, which is evident by an inability to provide certain vital cytokines such as IFNs and inflammatory cytokines [7]. Cord blood derived cells also have impaired expression of PRRs. For instance, cord NK cells have deficient TLR3 expression and are unable to respond to poly(I:C) and HSV activation, both in terms of cytokine secretion (IFN-γ) and cytotoxic capacity [8]. Furthermore, newborns also lack functionally experienced and expanded antigen-specific T- and B-cells [7]. B-cells from newborns have reduced strength of B-cell receptor signaling [9], and impaired CD40-mediated responses including antibody production and class switching [10]. In addition to being antigen-specific, B-cells also possess innate immune functions [11], such as the expression of TLRs that are expressed in both cord and adult B-cells [12]. In this paper, we have studied the expression and function of the DNA sensor AIM2 in in freshly isolated and *in vitro* activated cells derived from neonatal cord blood and adult peripheral blood. We found that AIM2 was preferentially expressed in adult B-cells, primarily by the mature CD27^+^ B-cell subset. Primary B-cells were induced to express AIM2 in response to IFN-γ (but not IFN-α), and refrained from AIM2 expression after cognate B-cell receptor engagement.

## Material and methods

### Study subjects

Fresh buffy coats of anonymized healthy blood donors and cord blood from anonymized healthy newborns born at gestation weeks 38–42 were obtained from Sahlgrenska University Hospital (Gothenburg, Sweden). In accordance to Swedish legislation section code 4§ 3p SFS 2003:460 (“Lag om etikprövning av forskning som avser människor”), no ethical approval was needed for buffy coats, since the buffy coats were provided anonymously and could not be traced back to a specific donor. All participants provided informed consent for blood donation. For the cord blood, all mothers were given oral information and gave oral consent to participate in the study. As no personal information or identity was recorded, no written consent or approval by the Human Research Ethics Committee was needed (Swedish law 2003: 460, paragraphs 4 and 13).

### Isolation and purification of adult and cord blood cells

Peripheral blood mononuclear cells (PBMCs) and cord blood mononuclear cells (CBMCs) were isolated by density gradient centrifugation on Ficoll-Hypaque PLUS (GE Healthcare Bio-Sciences AB, Uppsala, Sweden). B-cells, NK cells, CD4^+^ T-cells, CD8^+^ T-cells, plasmacytoid DC (pDC), myeloid DC (myDC), and monocytes (CD14^+^), were isolated by magnetic cell sorting. B-cells and NK cells were isolated by negative selection using a human B-cell isolation kit, (Invitrogen Dynal AS, Oslo, Norway), and a human NK cell isolation kit (Miltenyi Biotec GmbH, Bergisch Gladbach, Germany), respectively. CD4^+^ and CD8^+^ T-cells were purified using Dynal CD4- and CD8-positive isolation kits (Invitrogen Dynal AS, Oslo, Norway). MyDC, pDC and monocytes were purified by positive selection using the MicroBead kits for BDCA-1, BDCA-4 and CD14 (Miltenyi Biotec GmbH), respectively. CD27^+^ and CD27^-^ B-cells were isolated on a Synergy cell sorter (Sony Biotech), using monoclonal antibodies against CD19 (APC-H7) and CD27 (PE) (BD Biosciences, Heidelberg, Germany, cat no. 560177 and 340425, respectively). Antibody concentrations were 0.5 ul CD19 (APC-H7) per 1x10^6^ cells and 2 ul CD27 (PE) per 1x10^6^ cells. Cells were sorted as CD19^+^CD27^-^ and CD19^+^CD27^+^. The purity of CD19^+^CD27^-^ and CD19^+^CD27^+^ was 66–97% and 83–92%, respectively.

### In vitro activation of cells

B-cells and PBMC/CBMC were incubated for 2, 6, 12, 18 or 24 hours (2×10^5^ cells/200 μl in 96-well plates) in Iscove's medium (Sigma-Aldrich) (supplemented with l% glutamine, 1% gentamicin, 1% 2-mercaptoethanol, and 10% FBS) and Poly deoxyadenylic-thymidylic acid sodium salt (poly dA:dT) (10 ug/ml; Sigma-Aldrich), recombinant IFN-α (10 ng/ml; PBL Biomedical Laboratories, Piscataway, NJ, USA), recombinant IFN-γ (10 ng/ml; R&D Systems), anti-IgGAM (2.5 ug/ml; Jackson Laboratories, West Grove, US), CD40L and enhancer (1 ug/ml and 1 ug/ml, respectively; Enzo Life Sciences), anti-IgGAM+ CD40L and enhancer or Lipofectamine2000 Transfection Reagent (Invitrogen). Prior to stimulation, poly dA:dT was complexed with Lipofectamine2000 Transfection Reagent (Invitrogen), according to manufacturer’s instructions. Supernatants were collected after 4 and 24 hours of culture, and stored at -20°C until further use.

### AIM2 mRNA expression

The relative levels of AIM2 mRNA were analyzed in freshly isolated cells or in cells that had been activated *in vitro*. Briefly, cells were lysed with 350 μl lysis buffer (Qiagen, Hilden, Germany). Total RNA was extracted with an RNeasy Micro kit (Qiagen) and treated with DNase (Qiagen) to remove genomic DNA, using the QIACube (Qiagen). cDNA was prepared in a random hexamer-primed Superscript (Invitrogen, Carlsbad, CA, USA) RT reaction. The mRNA levels were determined by RT-PCR on a ViiA^™^ 7 Real-Time PCR System (TaqMan; Applied Biosystems, Foster City, CA, USA) using MicroAmp Optical 96-well reaction plates (Applied Biosystems). The primer-probe pairs were AIM2 (Hs00915710_m1), IFI16 (Hs00986757_m1), NLRP3 (Hs00918082_m1) and GAPDH (Hs99999905_m1) (TaqMan, Applied Biosystems). The samples (10 ng of cDNA) were run in duplicates in a 20-μl reaction mix (with TaqMan Universal PCR Master Mix; Applied Biosystems) using the comparative ΔΔCT method of relative quantification to calculate the differences in gene expression between control and antigen stimulated cells. As an endogenous control, GAPDH was used to correct for variations in sample loading. The samples were normalized to a standard consisting of a pool of cDNA from 10 adults that were set to 1.

### Flow cytometry

AIM2 protein expression was detected by FACS Verse (BD Biosciences), using the following monoclonal antibodies; AIM2 (PE, diluted 1:20, Biolegend, cat no. 652803), IgGκ (PE, diluted 1:20, Biolegend, cat no. 400135), CD19 (APC-Cy7, diluted 1:200, BD Biosciences, cat no. 560177) CD27 (Brilliant Violet 421, diluted 1:50, Biolegend, cat no. 356418), CD3 (Brilliant Violet 421, diluted 1:20, Biolegend, cat no. 300434) and CD56 (APC, diluted 1:5, BD Biosciences, cat no. 555518).

### Western blot

Western blot analysis was conducted with whole B cell lysates prepared from buffy coats. Protein extracts were prepared in hot 1% SDS and protein concentration was determined using the Pierce BCA protein assay kit. 25 ug of protein per sample were resolved on a 4–15% gradient SDS-PAGE gel and transferred onto a PVDF membrane. Nonspecific binding was blocked by soaking the membrane in a PBS-Tween (0.1%) buffer containing 5% bovine serum albumin for 1 h. AIM2 was detected using the rabbit monoclonal anti-AIM2 antibody diluted 1:500 (CST #12948). The membrane was then incubated with goat anti-rabbit IgG conjugated with horseradish peroxidase as a secondary antibody (diluted 1:10000). Imaging of protein bands was achieved by using enhanced chemiluminescence (clarity western ECL substrat, BioRad) and the ChemiDoc XRS system (BioRad).

### MitoSOX^™^

Adult B-cells were harvested after 24 hours of culture with lipofectamine or poly dA:dT, and stained for FACS using the following antibodies; CD19 (APC-Cy7, (BD Biosciences), CD27 (Brilliant Violet 421, BD Biosciences), and MiotSOX^™^ Red mitochondrial superoxide indicator (Invitrogen) according to manufacturer’s instructions. All samples were assayed on FACS Verse (BD Bioscience).

### Caspase-1 activity

Caspase-1 activity was measured in B-cells after 4 hours of culture with poly dA:dT or lipofectamine (control), using the FAM-FLICA^®^ Caspase 1 Assay Kit (ImmunoChemistry Technologies, MN, USA), according to manufacturer’s instructions. Cells were then stained for CD19 and CD27 expression (CD19 APC-Cy7, diluted 1:200, BD Biosciences, cat no. 560177 and CD27 Brilliant Violet 421, diluted 1:50, Biolegend, cat no. 356418), and assayed on FACS Verse.

### Cytokine detection

IL-1β and IFN-α secretion in supernatants from cell cultures were analyzed using a Duo Set ELISA kit (R&D Systems) and a VeriKine human IFN-α ELISA kit (PBL assay science, NJ, US), respectively, both according to manufacturer’s instructions.

### Statistics

Statistics were calculated using one-way ANOVA followed by Tuckey’s or Dunnett’s multiple comparison test, Students paired t-test, Mann–Whitney U-test, and Wilcoxon matched-pairs signed rank test (PRISM 6.0^®^ Graph Pad Software Inc., San Diego, CA).

## Results

### AIM2 is preferentially expressed in adult B-cells

**Fig 1:**
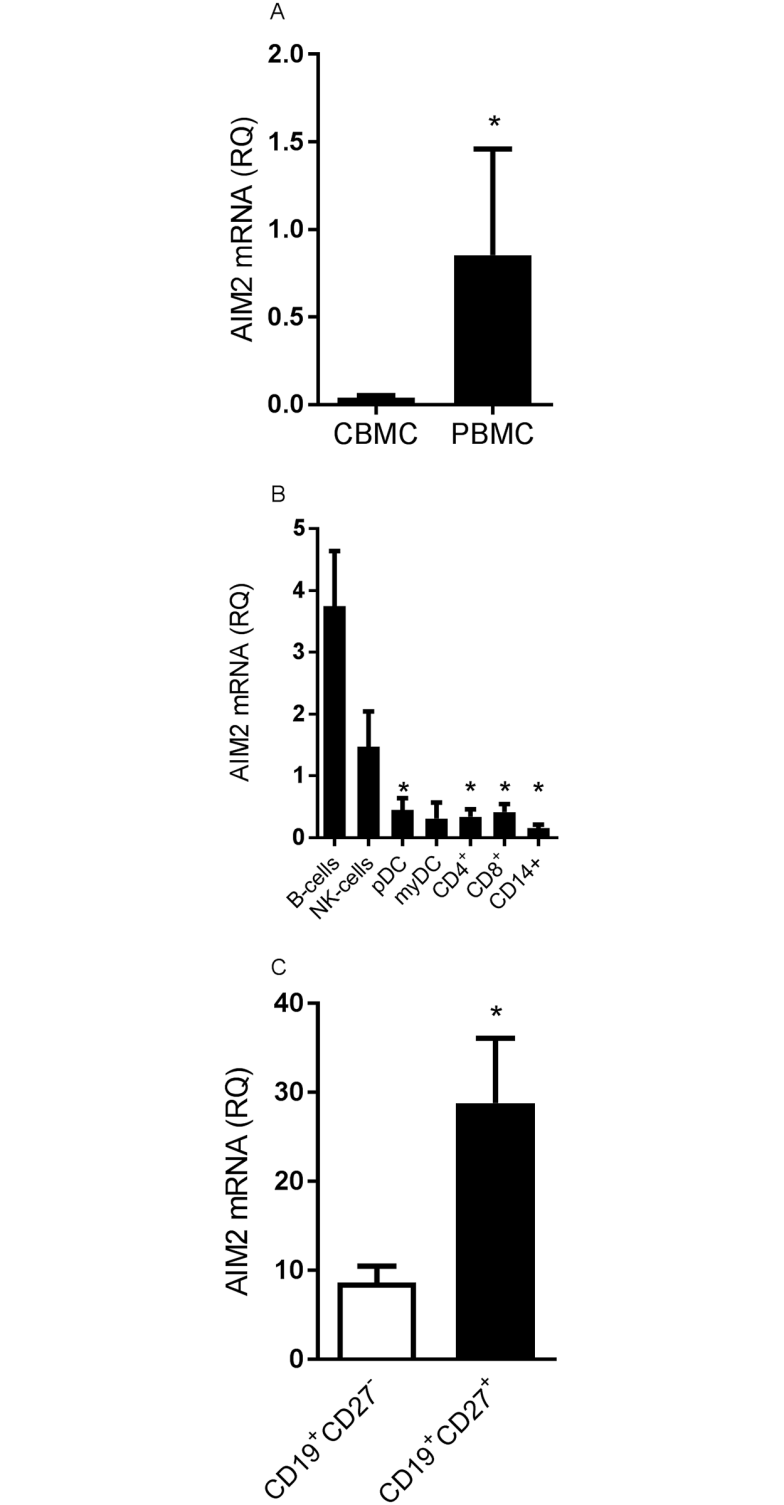
AIM2 mRNA is expressed in adult B-cells. AIM2 mRNA expression was measured in PBMC and CBMC (A), and in various cell types (i.e. B-cells, CD4^+^ and CD8^+^ T-cells, monocytes, NK cells, plasmacytoid dendritic cells and myeloid dendritic cells) that were extracted using magnetic separation (B), and in B-cells that were sorted by flow cytometry using CD19 and CD27 antibodies (C). The relative quantification (RQ) was calculated by the AIM2 versus the GAPDH mRNA ratio in adult (A-C) and cord blood derived cells (A). A pool of 10 PBMCs was used as a calibrator sample and set to a value of 1. Data is expressed as the mean AIM2 mRNA expression +SEM from 2–7 individuals/group. Statistics were calculated using Mann–Whitney U-test (A), ordinary one-way ANOVA and Tuckey’s multiple comparison test (B) (* = p<0.05 compared to B-cells), and Wilcoxon matched-pairs signed-rank test (C). * = p<0.05.

**Fig 2:**
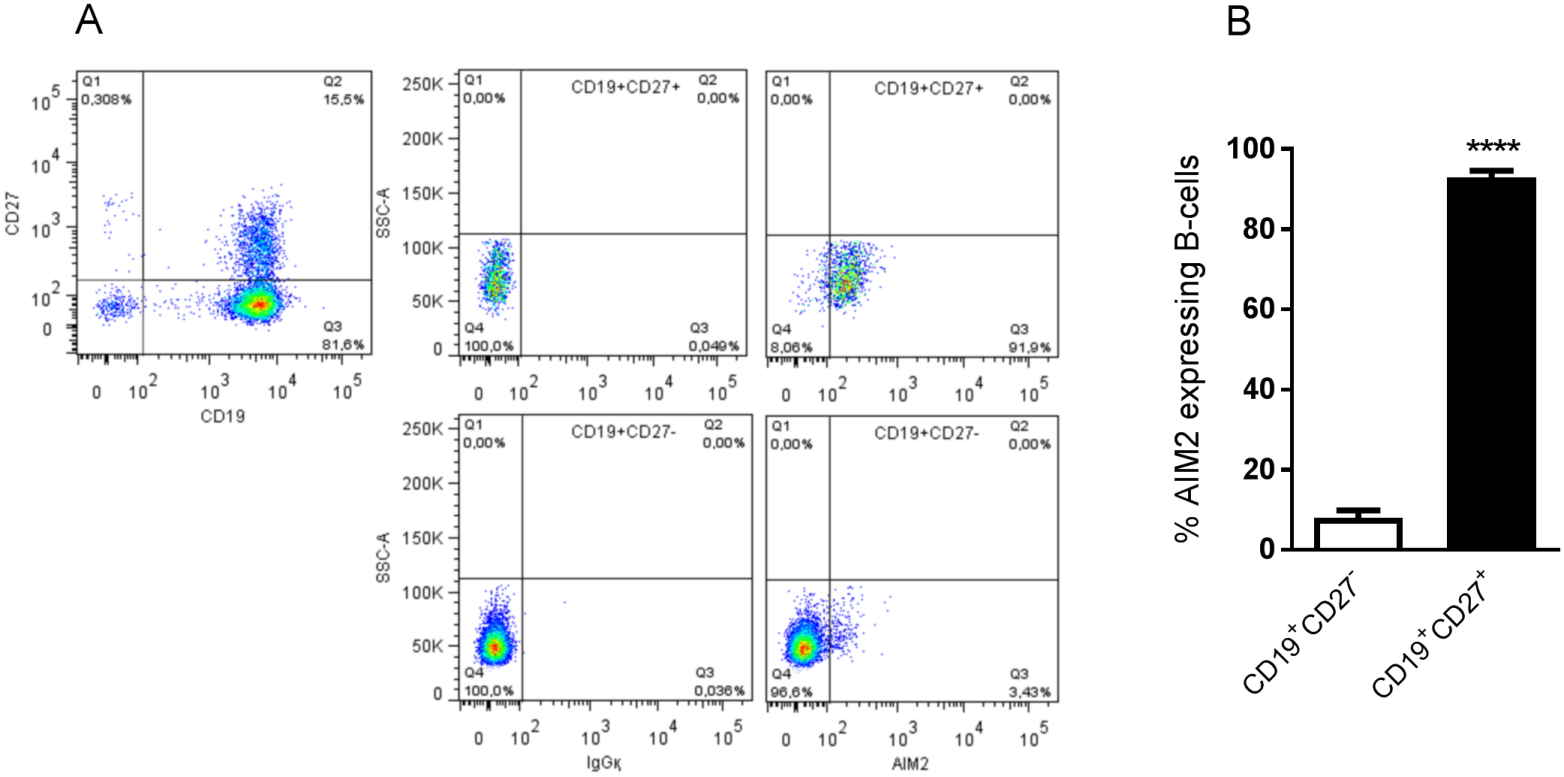
AIM2 protein is expressed in adult CD27^+^ B-cells. Magnetically extracted adult B-cells were stained for FACS-analysis using CD19, CD27 and AIM2 antibodies. Data is presented as FACS-plots of B-cells expressing CD19 and CD27 (left plot), and IgGқ (middle panels) and AIM2 (right panels) expressing CD19^+^CD27^+^ (top panels) and CD19^+^CD27^-^ (bottom panels) B-cells from one representative experiment (A), and frequency of AIM2 expressing CD19^+^CD27^+^ (filled bars) and CD19^+^CD27^-^ (empty bars) B-cells from 4 individuals (B). Data is expressed as the mean expression +SEM of AIM2. Statistics were calculated using paired t-test; **** = p<0.0001.

We compared the expression of AIM2 in freshly isolated PBMC and CBMC. CBMC expressed low levels of AIM2 mRNA, whereas the AIM2 mRNA expression in PBMC was significantly higher (Fig 1A). A more detailed analysis of different mononuclear cells (B-cells, NK cells, CD4^+^ T-cells, CD8^+^ T-cells, pDC, myDC and monocytes) revealed that AIM2 was preferentially expressed in adult B-cells (Fig 1B), whereas other mononuclear cells derived from both cord and adult blood expressed no, or low, levels of AIM2 (Fig 1B and S2 Fig). Both adult and cord B-cells expressed comparable levels of IFI16 and NLRP3 mRNA (S3 Fig). To further assess the AIM2 mRNA expression in B-cells, we sorted cells derived from adult blood as naïve CD19^+^CD27^-^ and memory CD19^+^CD27^+^ cells. CD19^+^CD27^+^ B-cells expressed significantly higher levels of AIM2 mRNA compared to CD19^+^CD27^-^ B-cells (Fig 1C). To confirm the AIM2 mRNA expression in B-cells on protein level, we stained adult B-cells for FACS analysis. In line with the mRNA expression, we found that the AIM2 protein was primarily expressed by CD19^+^CD27^+^ B-cells but not by CD19^+^CD27^-^ B-cells (Fig 2A and 2B). 92% (range: 90–98%) of the CD19^+^CD27^+^ B-cells expressed AIM2 compared to only 8% (range: 3.4–8.7%) of the CD19^+^CD27^-^ B-cells (p<0.0001) (Fig 2B). The AIM2 protein in B-cells was also visible as a 37 and a 53 kDa band on Western blot (S4 Fig). NK cells expressed somewhat higher levels of AIM2 mRNA, as compared to the other cell types (Fig 1B). This was however not reflected on a protein level, as no AIM2 protein was detected in NK cells (S5 Fig).

### IFN-γ induce AIM2 mRNA expression in adult and cord blood B-cells

**Fig 3:**
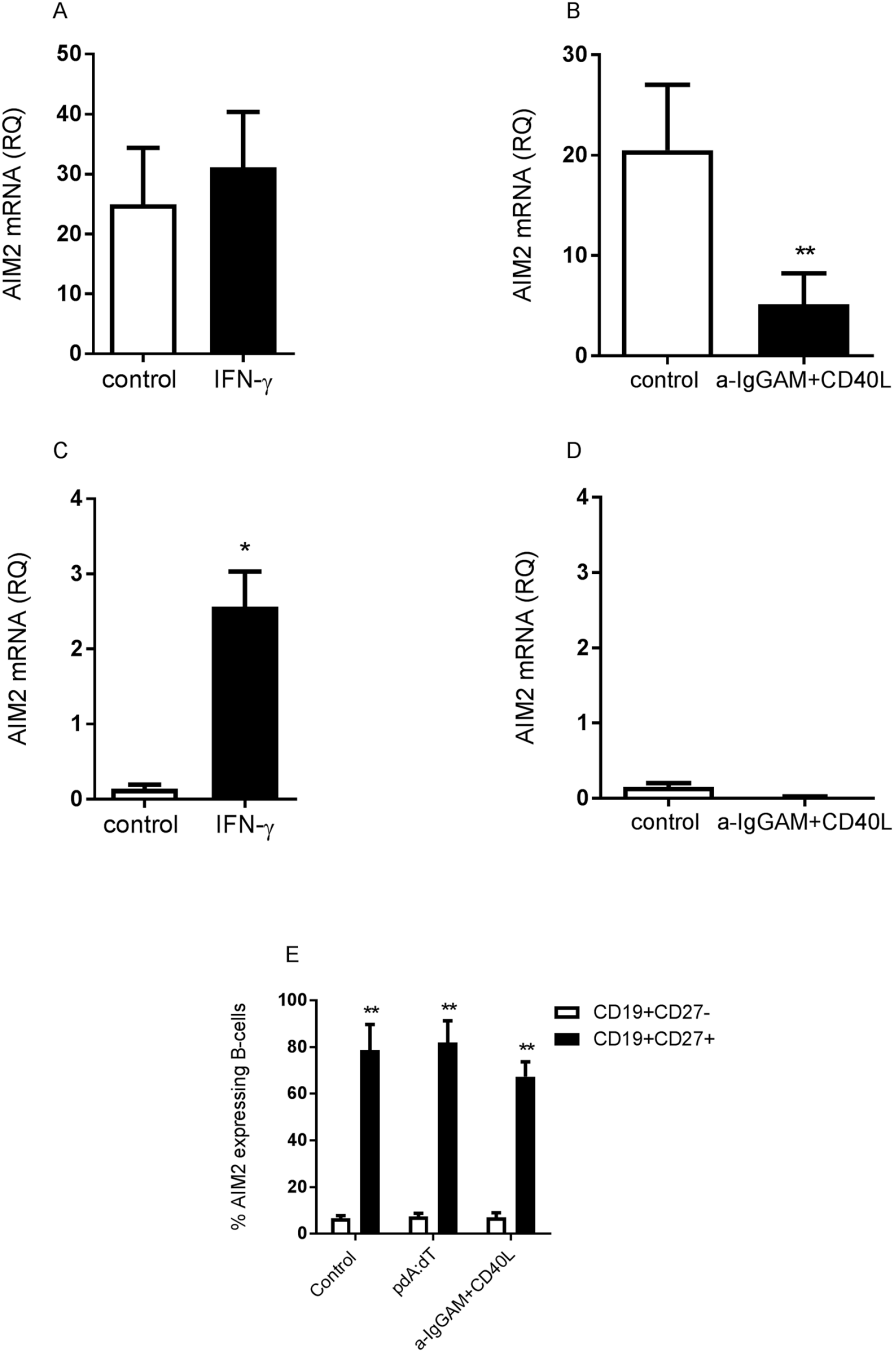
*In vitro* stimulation with IFN-γ induces AIM2 mRNA expression in B-cells. Adult (A, B and E) and cord (C and D) B-cells were collected after 24 hours of culture with IFN-γ (A and C), α-IgG, A and M + CD40L (B and D), poly dA:dT (E) or lipofectamine (control) (A-E), and assessed for AIM2 mRNA expression (A-D) or AIM2 protein expression (E). Data is expressed as the mean expression +SEM from 3–9 individuals. Statistics were calculated using Students paired t-test; * = p<0.05; ** = p<0.01.

To asses if AIM2 expression could be induced via autokrine activation of AIM2, cytokines or B-cell receptor engagement, we cultured human B-cells derived from adult and cord blood with the AIM2 ligand poly dA:dT, the cytokines IFN-α and IFN-γ or with anti-IgGAM plus CD40L for 24 hours and measured their AIM2 mRNA expression. INF-γ was the only cytokine that was able to increase the AIM2 mRNA expression in B-cells from adults when compared to control stimulated cells (Fig 3, S1 and S6 Figs) (p<0.02). Furthermore, adult B-cells activated by anti-IgGAM+CD40L expressed significantly lower levels of AIM2 mRNA, compared to control stimulated cells (Fig 3B) (p<0.01). AIM2 mRNA expression was not significantly reduced by IgGAM or CD40L stimulation alone, even though there was a tendency of reduced expression in both IgGAM and CD40L stimulated B-cells (S7 Fig). Similar to adult B-cells, cord B-cells failed to upregulate AIM2 mRNA in response to poly dA:dT, IFN-α or anti-IgGAM plus CD40L (Fig 3D and S1 Fig), but cord blood B-cells showed a 16-fold increase of AIM2 mRNA expression following IFN-γ exposure (Fig 3C) (p<0.05). The expression of AIM2 mRNA in IFN-γ stimulated cord B-cells were still considerably lower (15 times) compared to the levels of AIM2 mRNA in IFN-γ stimulated adult B-cells. When we analyzed AIM2 protein expression in the cultured cells, we found that CD27^+^ B-cells were the main contributors to the AIM2 expression. CD27^+^ adult B-cells expressed 9–12 times more AIM2 protein, as compared to CD27- adult B-cells (Fig 3E) (p<0.01). However, none of the stimuli used (i.e. poly dA:dT or IgGAM+CD40L) were able to enhance the frequency of AIM2 expressing B-cells (Fig 3E). Additionally, we also asked whether stimulation of B-cells with poly dA:dT could induce the expression of the other ALR, i.e. IFI16. However, B-cells did not upregulate the expression of IFI16 mRNA in response to the synthetic DNA (S8 Fig).

### Synthetic DNA induce secretion of IL-1β in *in vitro* stimulated B-cells

**Fig 4:**
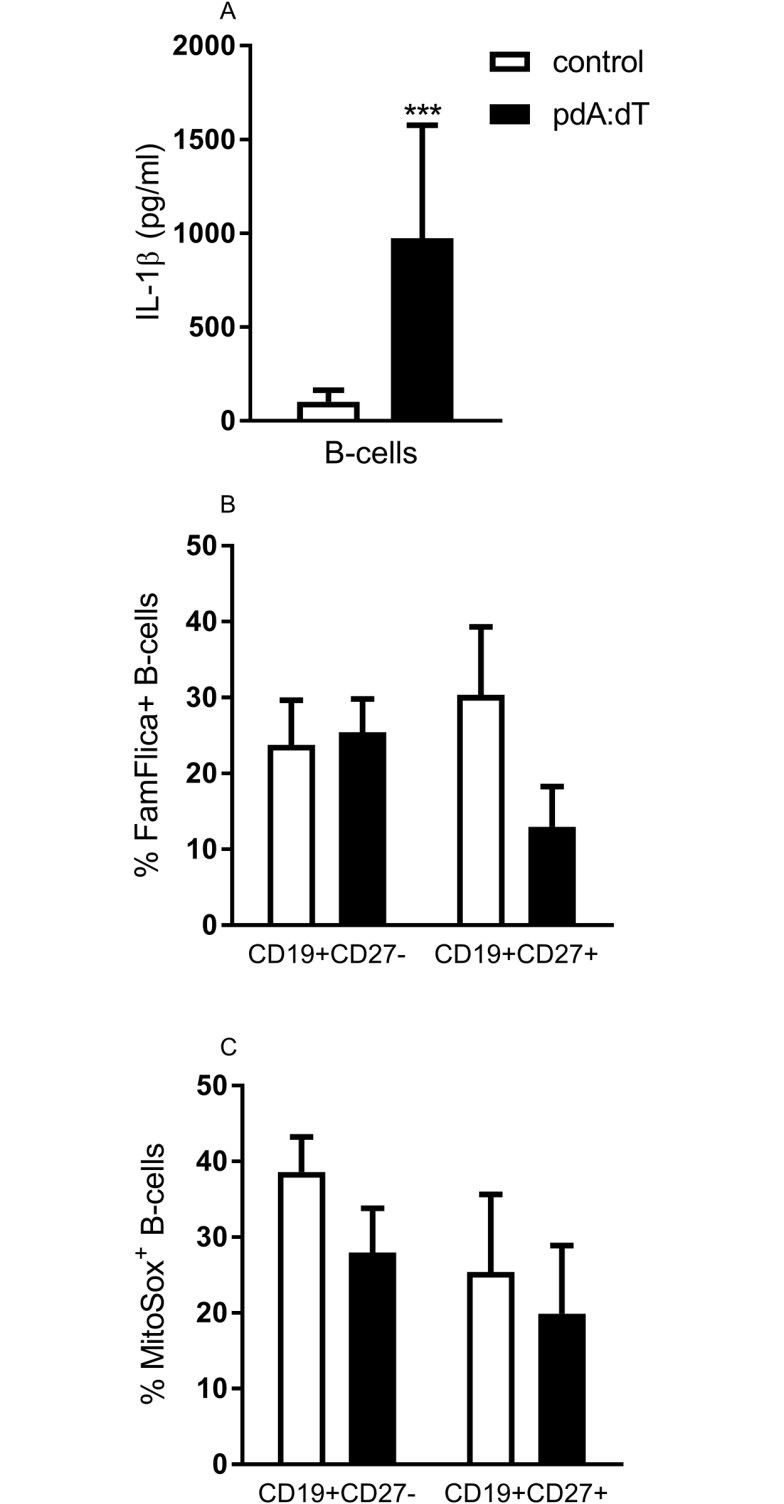
*In vitro* stimulated B-cells secrete IL-1β in response to synthetic DNA. B-cells derived from adult blood were cultured with lipofectamine or poly dA:dT and assayed for IL-1β secretion (A), expression of Fam-flica (B) and expression of MitoSOX (C). IL-1β secretion was measured in supernatants after 4 hours of culture (A). Expression of Fam-Flica (B) and mitochondrial superoxide (C) was analyzed by FACS after 4 (B) and 24 (C) hours of culture. Data represent the mean +SEM from 3–14 individuals. Statistics were calculated using Wilcoxon matched-pairs signed rank test; *** = p<0.001.

To assess the function of AIM2 expression in B-cells, we measured IL-1β secretion, cell death and mitochondrial superoxide production in cells cultured with poly dA:dT. The IL-1β secretion was significantly increased (9-fold) in poly dA:dT stimulated adult B-cells compared to control stimulated B-cells (Fig 4A) (p<0.001). To assess if the AIM2 expression in adult B-cells had an effect on cell death, we stained cultured B-cells for active caspase-1 (i.e. Fam-Flica) (Fig 4B) or mitochondrial superoxide (Fig 4C). Stimulation with poly dA:dT did not induce increased frequencies of CD27^-^ or CD27^+^ B-cells that expressed active caspase-1 (Fig 4B). Similarly, stimulation of B-cells with poly dA:dT did not affect the release of mitochondrial superoxide, as the frequency of MitoSOX positive B-cells did not differ between control and poly dA:dT stimulated B-cells (Fig 4C).

## Discussion

In this paper we show that adult but not cord blood mononuclear cells express AIM2. In adults, B-cells and in particular the CD27 positive subset was the main contributors to the AIM2 expression. These cells further upregulated their AIM2 mRNA expression in response to IFN-γ. In cord B-cells, the steady-state levels of AIM2 were low but could be induced upon exposure to IFN-γ.

We have previously shown that newborns have impaired innate immune responses, where NK cells from cord blood have reduced expression of TLR3 as well as deficient TLR3 mediated IFN-γ secretion and cytotoxic capacity [8]. In the current study we show that mononuclear cells from newborns also lack the DNA sensor AIM2. In our hands, none of the investigated cell types (i.e. CBMC, B-cells, CD4^+^ and CD8^+^ T-cells, monocytes, NK cells, plasmacytoid dendritic cells and myeloid dendritic cells) expressed AIM2 mRNA. Similar to the cord blood derived cells, AIM2 expression was absent also in the majority of the analyzed cell types derived from adult peripheral blood, with the exception of B-cells. Previous studies have shown that AIM2 is expressed in B-cells derived from adult peripheral blood at steady state, whereas monocytes and PBMC express no or very low levels of AIM2 mRNA [13], which is in line with our findings. We found that AIM2 was primarily expressed by the “mature” CD27^+^ B-cells, and to a lesser extent in “naïve” CD27^-^ B-cells. Thus, the poor expression of AIM2 mRNA in neonatal B-cells is most likely due to the lack of antigen experienced CD27^+^ B-cells in cord blood [14]. Of note, the expression of AIM2 mRNA may also vary with gender [15].

Expression of AIM2 can be induced in different cell types by both double stranded DNA and IFNs. We activated purified B-cells derived from cord and adult blood *in vitro* with the AIM2 ligand poly dA:dT and IFNs (IFN-α and IFN-γ). Stimulation with IFN-γ did induce AIM2 mRNA expression in both cord and adult cells, which is in line with previous studies in other cell types, i.e. THP-1 cells and human keratinocytes [13,16]. However, the IFN-γ induced AIM2 mRNA expression in cord B-cells were modest compared the steady state levels in adult B-cells. B-cells did not upregulate AIM2 transcription in response to type I IFN or to the AIM2 ligand poly dA:dT. This is in contrast to previous observations, showing that AIM2 can be induced in response to both IFN-α and poly dA:dT [1–3,5,13]. However, the previous studies were conducted in cell lines or in murine cells/models, which are considerably different from freshly isolated human (B-) cells. Furthermore, the impact of type 1 IFNs on AIM2 expression and inflammasome activation is not clear cut, as type 1 IFN has been shown to inhibit inflammasome activation in murine cells [17]. Interestingly, adult B-cells down regulated the expression of AIM2 upon activation through the B-cell receptor and CD40. This suggests that AIM2 is expressed in resting memory B-cells, but is down-modulated upon activation following re-exposure to cognate antigen. We can only speculate in why AIM2 is down modulated upon B-cell receptor activation. Given that AIM2 can suppress cell proliferation [18–21], down modulation of AIM2 may be of importance to allow the B-cell to enter cell division (i.e. the normal response that is induced upon stimulation of the B-cell receptor). This remains to be further investigated.

It has previously been shown that activation of AIM2 leads to the release of mature IL-1β and caspase-1 activity [3,5]. We show that primary B-cells that were stimulated with synthetic DNA did secrete IL-1β, albeit at moderate levels. The release of IL-1β was however not correlated to caspase-1 activity, as it remained unaffected upon stimulation with synthetic DNA. The release of IL-1β can occur independently of inflammasome activation, at least in neutrophils, where other enzymes than caspase-1 induce secretion of IL-1β [22]. Human B-cells has been shown to lack active caspase-1 at steady state [13]. We found low levels of caspase-1 activity after 4 hours of *in vitro* culturing. The differences may be due to the different methods used (i.e. immunoblotting or Fam-Flica), or by the events occurring during in vitro culturing.

In conclusion, we show that CD27 positive B-cells are the main cell type expressing AIM2 in adults, whereas cord B-cells was devoid of AIM2 mRNA. We also show that neither type I IFN nor synthetic double stranded DNA could induce AIM2 transcription, whereas type II IFN did promote AIM2 expression in both cord and adult B-cells.

## Supporting information

S1 Fig AIM2 expression in in vitro activated B-cells. Adult (A and B) and cord (C and D) B-cells were assessed for AIM2 mRNA expression after 24 hours of culture with poly dA:dT (A and C), IFN-α (B and D), or lipofectamine (control) (A-D). Data is expressed as the mean expression +SEM from 5–14 individuals.
(TIF)

S2 Fig AIM2 expression in cord mononuclear cells. AIM2 mRNA expression was measured in freshly isolated cord blood derived cells (i.e. B-cells, CD4^+^ and CD8^+^ T-cells, monocytes, NK cells, plasmacytoid dendritic cells and myeloid dendritic cells) that were extracted using magnetic separation. The relative quantification (RQ) was calculated by the AIM2 versus the GAPDH mRNA ratio, and a pool of 10 PBMCs was used as a calibrator sample and set to a value of 1. Data is expressed as the mean AIM2 mRNA expression +SEM from 2-7donors.
(TIF)

S3 Fig No difference between cord and adult B-cells in IFI16 or NLRP3 mRNA expression. IFI16 (A) and NLRP3 (B) mRNA expression was measured in freshly isolated cord and adult B-cells. The relative quantification (RQ) was calculated by the IFI16 (A) or the NLRP3 (B) versus the GAPDH mRNA ratio in cord or adult B-cells. A pool of 10 PBMCs was used as a calibrator sample and set to a value of 1. Data is expressed as the mean AIM2 mRNA expression +SEM from 3 individuals/group. Statistics were calculated using students t-test.
(TIF)

S4 Fig AIM2 expression in B-cells detected by western blot. Cell extracts from freshly isolated adult B-cells were analyzed by western blot using an antibody specific to AIM2. Different forms of the AIM2 protein are visible as a 37 and a 53 kDa band.
(TIF)

S5 Fig AIM2 is not expressed in NK cells. PBMC were stained for FACS-analysis using CD3, CD56 and AIM2 antibodies. Data is presented as FACS-plots of PBMC expressing CD3 and CD56 (left panel), and CD3^-^CD56^+^ cells expressing AIM2 (right panel) from one representative donor out of three.
(TIF)

S6 Fig AIM2 mRNA expression at different time points after IFN-γ exposure. Adult B-cells were assessed for AIM2 mRNA expression after 6, 12, 18 and 24 hours of culture with IFN-γ (filled circles) or medium alone (empty circles). Data is expressed as the mean expression +SEM from 3 individuals.
(TIF)

S7 Fig AIM2 mRNA expression is reduced in response to anti-IgGAM and CD40L stimulation. Adult B-cells were assessed for AIM2 mRNA expression after 24 hours of culture with α-IgGAM (bars with horizontal lines), CD40L (bars with vertical lines), α-IgGAM + CD40L (black bars) or medium alone (white bars). Data is expressed as the mean expression +SEM from 3 individuals. Statistics were calculated using one way ANOVA followed by Dunnett’s multiple comparison test. * = p<0.05.
(TIF)

S8 Fig Stimulation with poly dA:dT does not upregulate IFI16 mRNA expression. Adult B-cells were assessed for IFI16 mRNA expression after 24 hours of culture with poly dA:dT or lipofectamine (control). Data is expressed as the mean expression +SEM from 3 individuals. Statistics were calculated using students paired t-test.
(TIF)
