# Supplementary figures and images for: Maturation-dependent expression of AIM2 in human B-cells
Source: PLoS One. 2017 Aug 15;12(8):e0183268. doi: 10.1371/journal.pone.0183268 (PMC5557365; doi:10.1371/journal.pone.0183268)

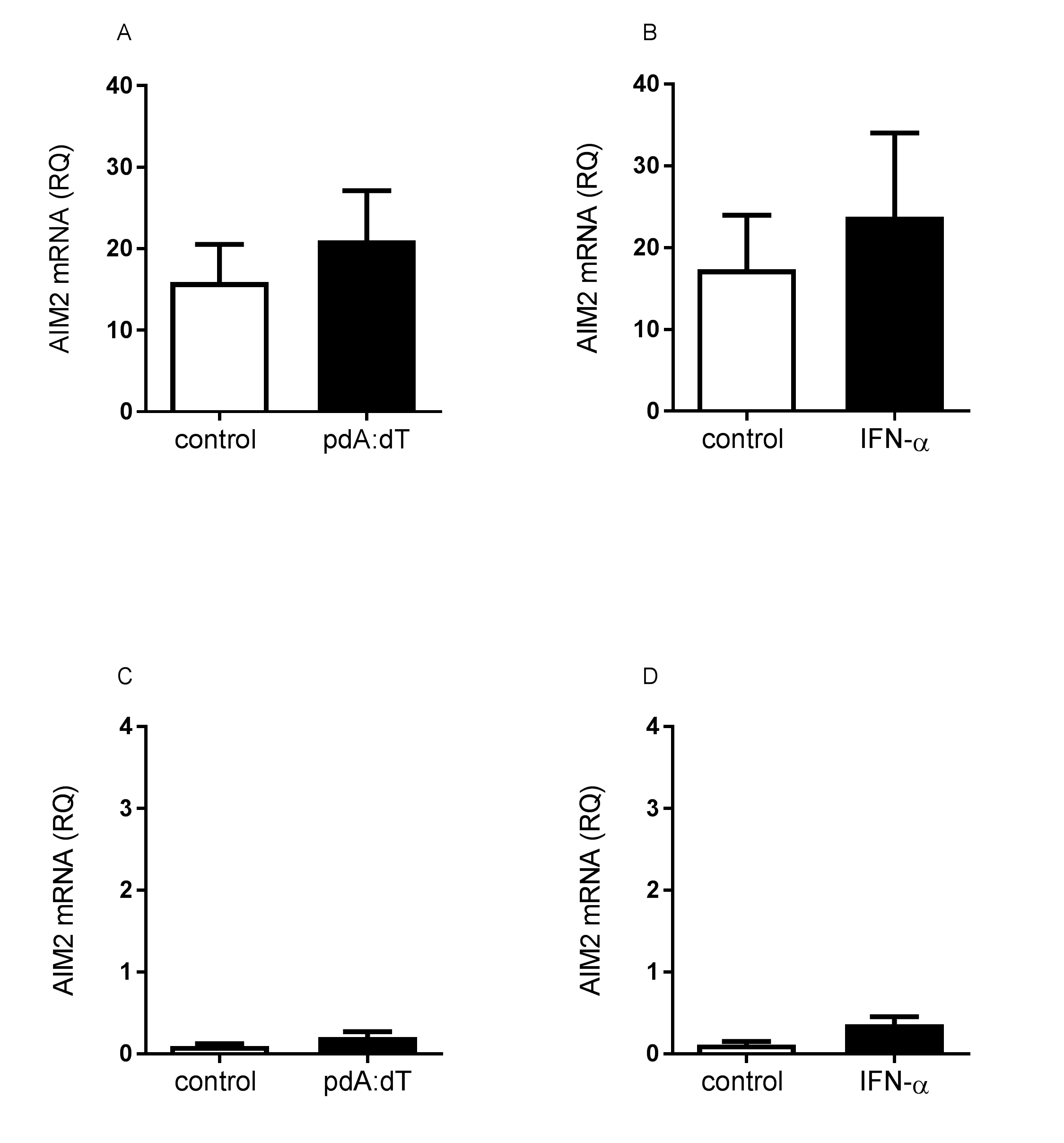

Supplement: S1 Fig — Adult (A and B) and cord (C and D) B-cells were assessed for AIM2 mRNA expression after 24 hours of culture with poly dA:dT (A and C), IFN-α (B and D), or lipofectamine (control) (A-D). Data is expressed as the mean expression +SEM from 5–14 individuals. (TIF) [file pone.0183268.s001.tif]

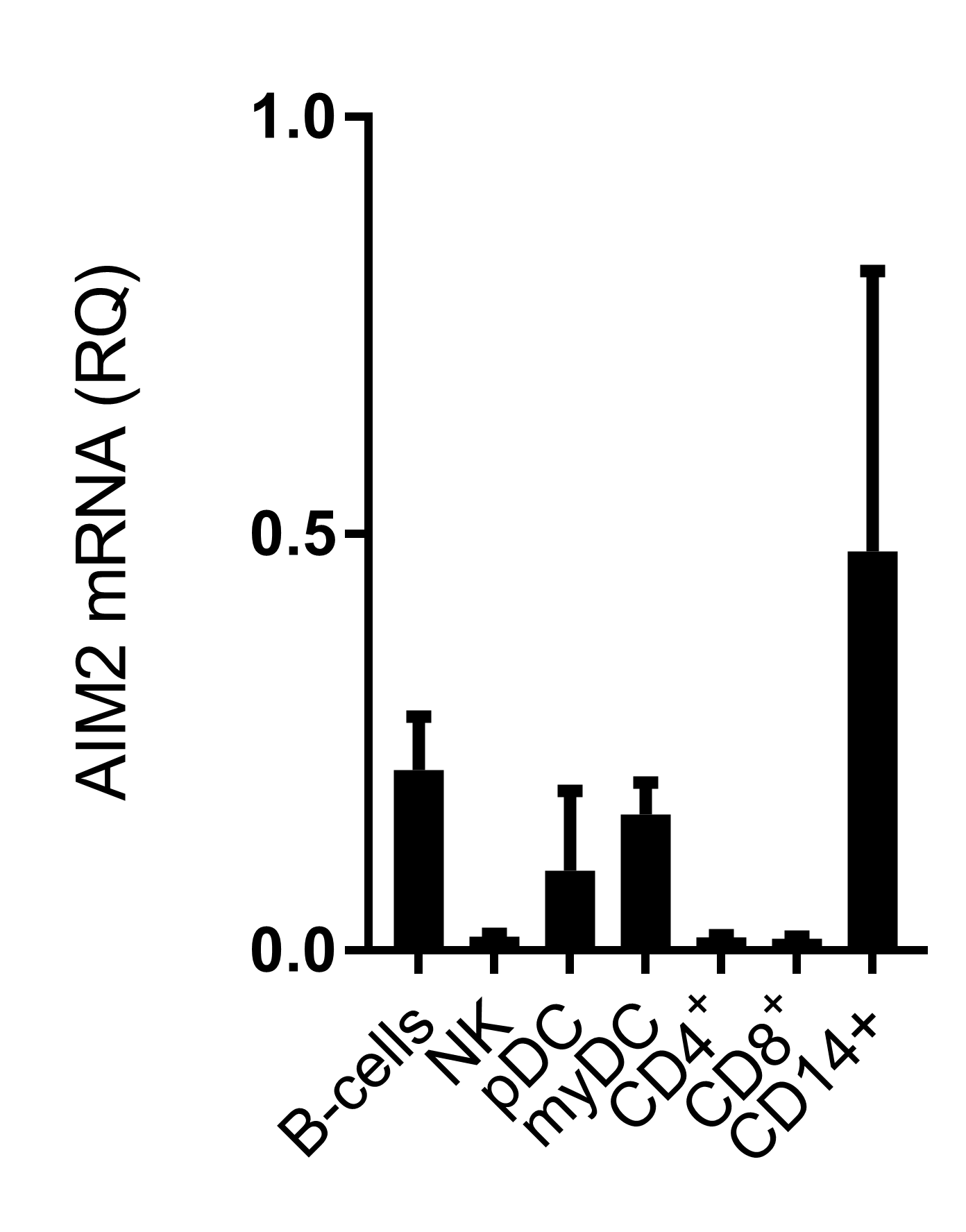

Supplement: S2 Fig — AIM2 mRNA expression was measured in freshly isolated cord blood derived cells (i.e. B-cells, CD4+ and CD8+ T-cells, monocytes, NK cells, plasmacytoid dendritic cells and myeloid dendritic cells) that were extracted using magnetic separation. The relative quantification (RQ) was calculated by the AIM2 versus the GAPDH mRNA ratio, and a pool of 10 PBMCs was used as a calibrator sample and set to a value of 1. Data is expressed as the mean AIM2 mRNA expression +SEM from 2-7donors. (TIF) [file pone.0183268.s002.tif]

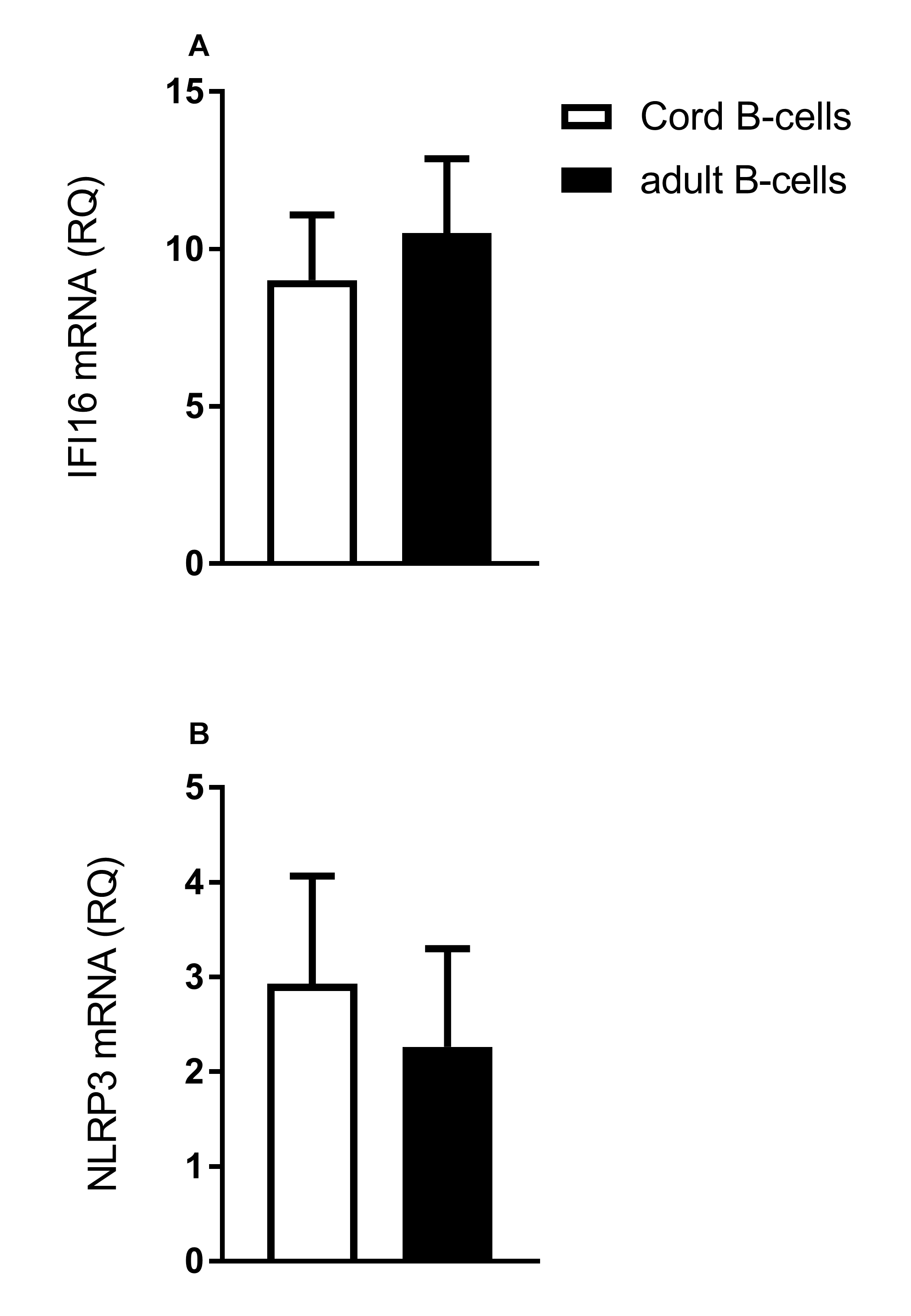

Supplement: S3 Fig — IFI16 (A) and NLRP3 (B) mRNA expression was measured in freshly isolated cord and adult B-cells. The relative quantification (RQ) was calculated by the IFI16 (A) or the NLRP3 (B) versus the GAPDH mRNA ratio in cord or adult B-cells. A pool of 10 PBMCs was used as a calibrator sample and set to a value of 1. Data is expressed as the mean AIM2 mRNA expression +SEM from 3 individuals/group. Statistics were calculated using students t-test. (TIF) [file pone.0183268.s003.tif]

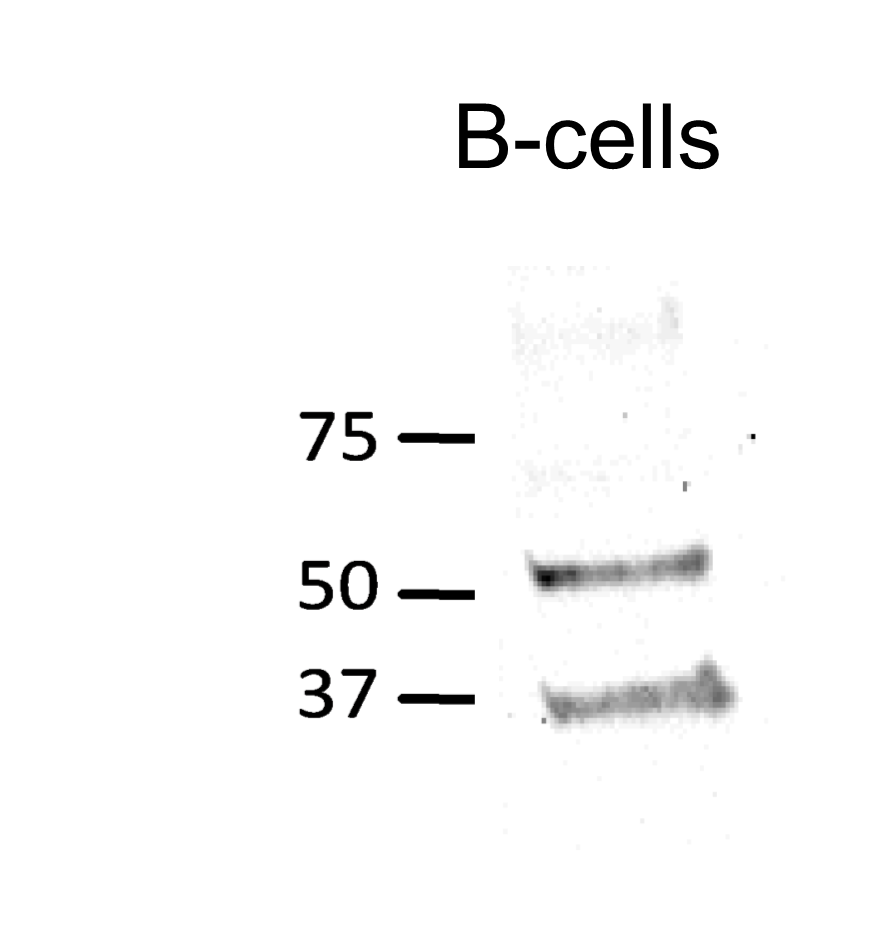

Supplement: S4 Fig — Cell extracts from freshly isolated adult B-cells were analyzed by western blot using an antibody specific to AIM2. Different forms of the AIM2 protein are visible as a 37 and a 53 kDa band. (TIF) [file pone.0183268.s004.tif]

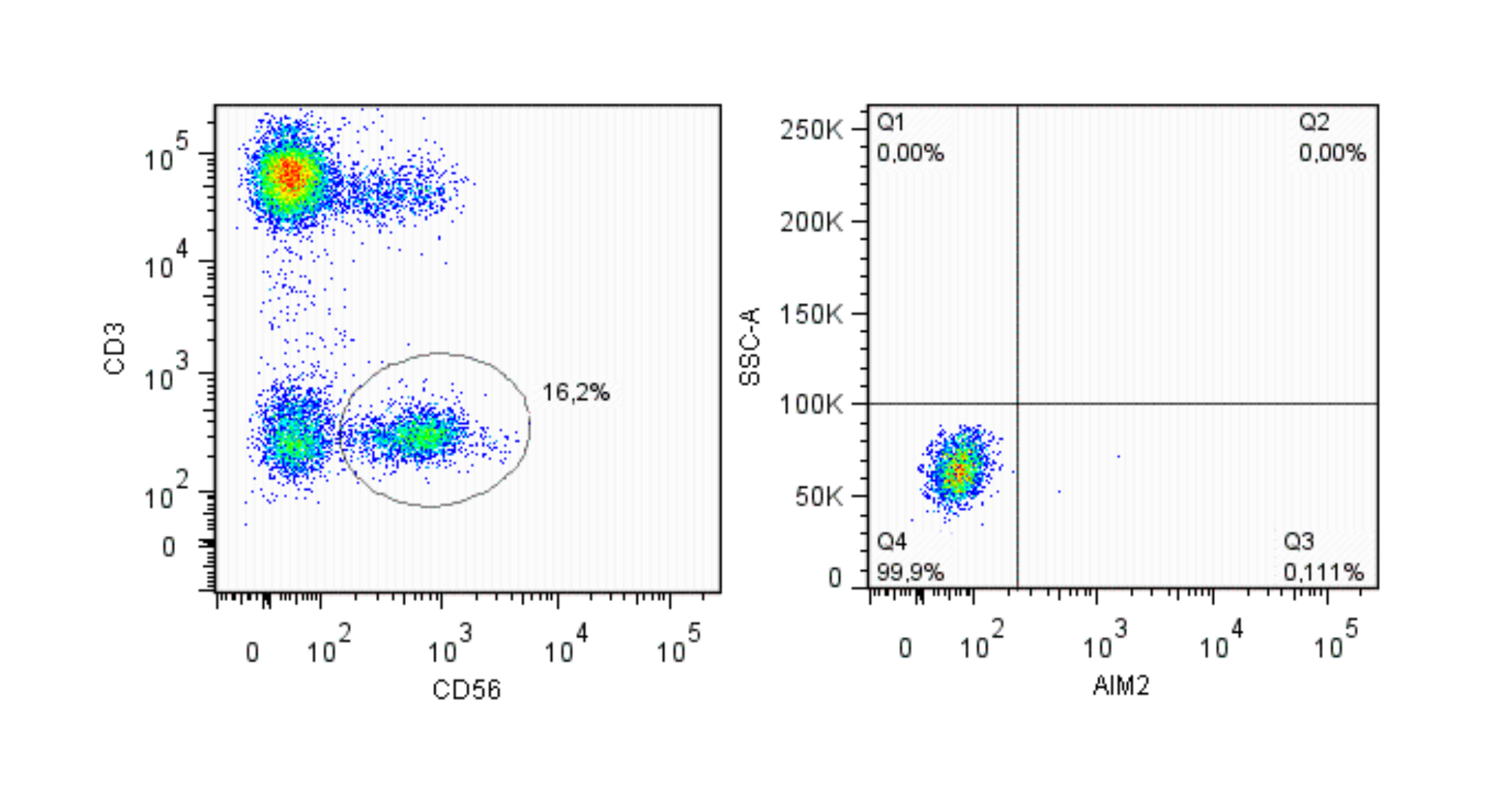

Supplement: S5 Fig — PBMC were stained for FACS-analysis using CD3, CD56 and AIM2 antibodies. Data is presented as FACS-plots of PBMC expressing CD3 and CD56 (left panel), and CD3-CD56+ cells expressing AIM2 (right panel) from one representative donor out of three. (TIF) [file pone.0183268.s005.tif]

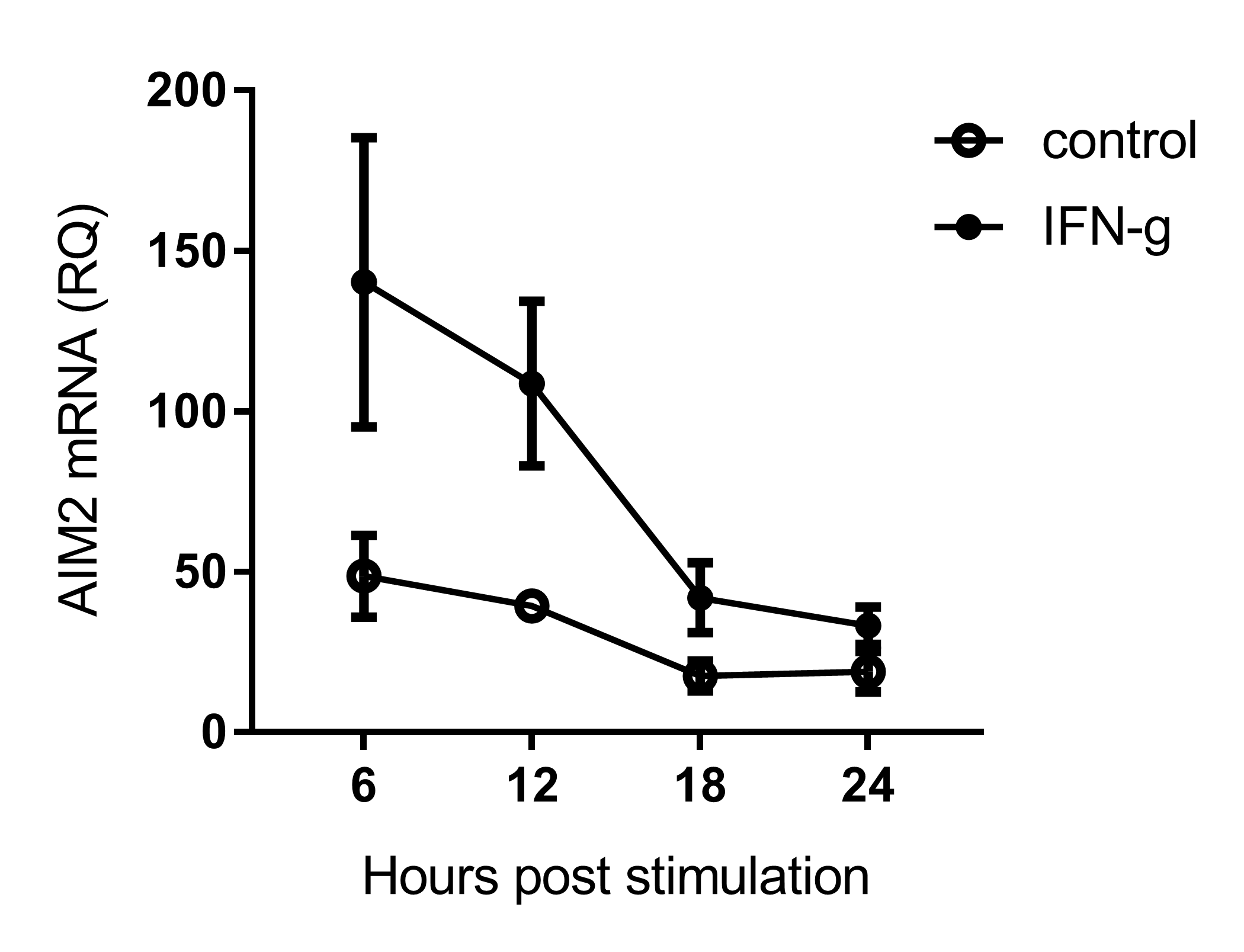

Supplement: S6 Fig — Adult B-cells were assessed for AIM2 mRNA expression after 6, 12, 18 and 24 hours of culture with IFN-γ (filled circles) or medium alone (empty circles). Data is expressed as the mean expression +SEM from 3 individuals. (TIF) [file pone.0183268.s006.tif]

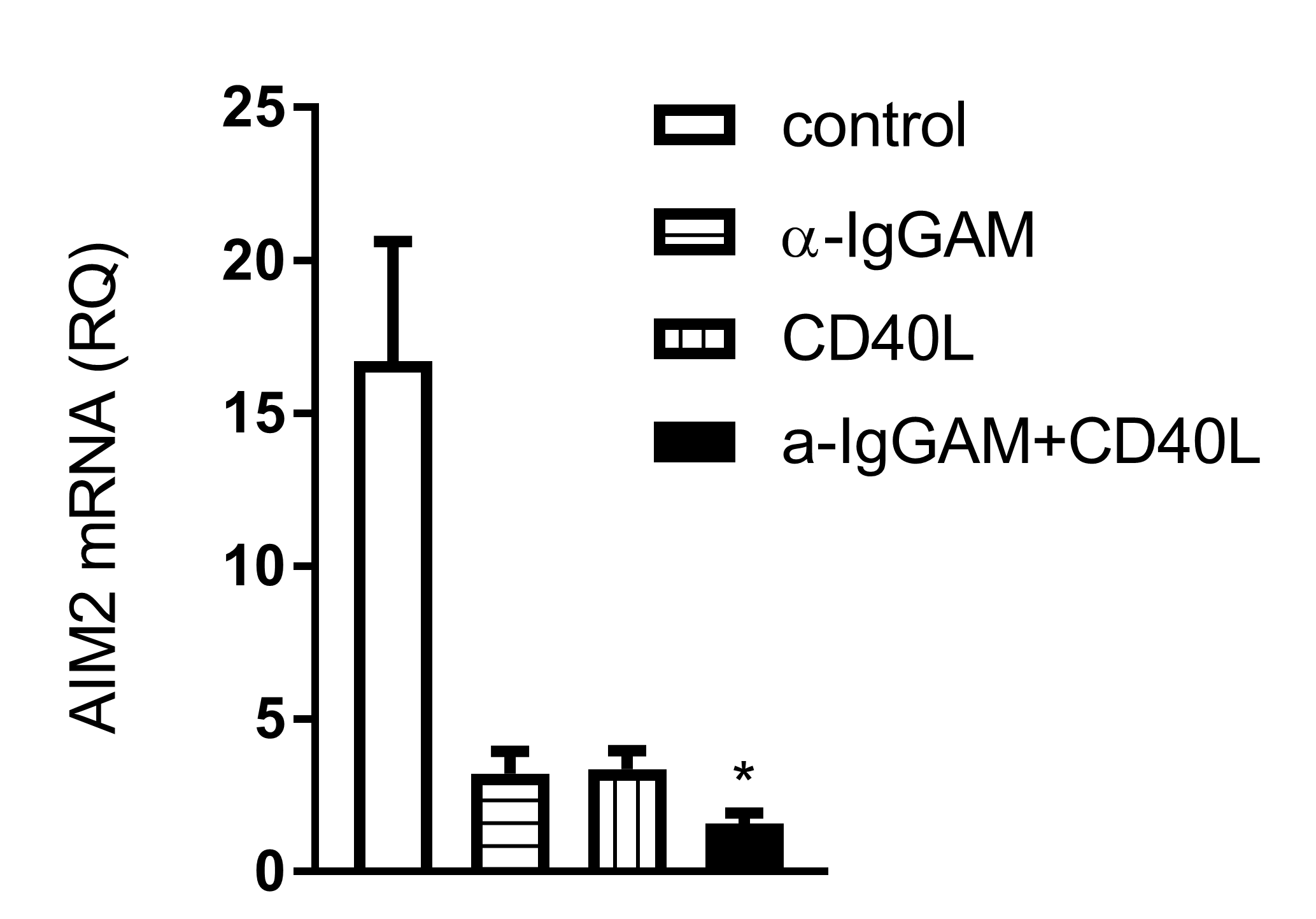

Supplement: S7 Fig — Adult B-cells were assessed for AIM2 mRNA expression after 24 hours of culture with α-IgGAM (bars with horizontal lines), CD40L (bars with vertical lines), α-IgGAM + CD40L (black bars) or medium alone (white bars). Data is expressed as the mean expression +SEM from 3 individuals. Statistics were calculated using one way ANOVA followed by Dunnett’s multiple comparison test. * = p<0.05. (TIF) [file pone.0183268.s007.tif]

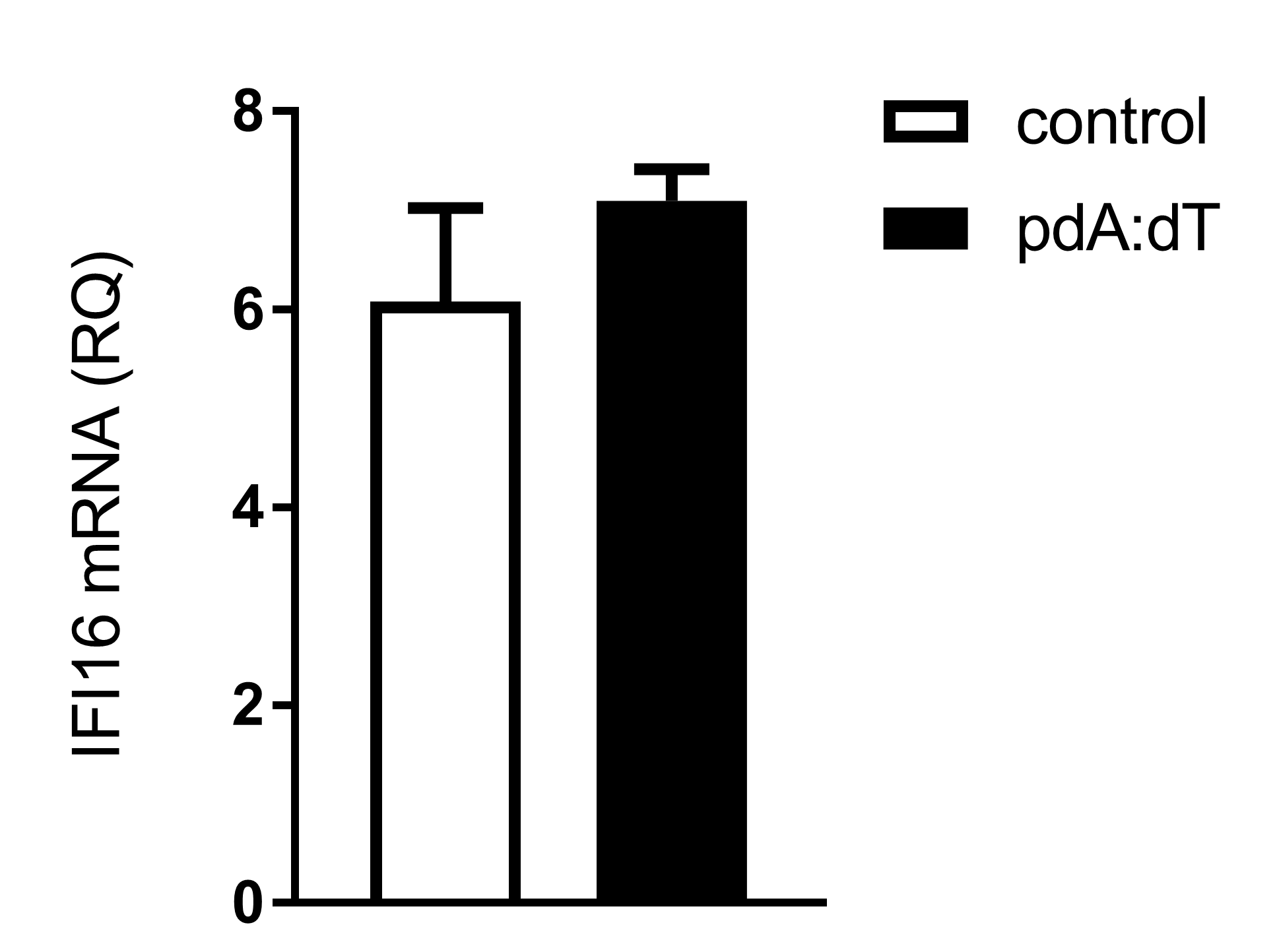

Supplement: S8 Fig — Adult B-cells were assessed for IFI16 mRNA expression after 24 hours of culture with poly dA:dT or lipofectamine (control). Data is expressed as the mean expression +SEM from 3 individuals. Statistics were calculated using students paired t-test. (TIF) [file pone.0183268.s008.tif]
